# Thyrotoxic Myopathy with Nonspecific Ophthalmopathy in a Two-Year-Old Child: Case Report and Literature Review

**DOI:** 10.3390/jcm13206180

**Published:** 2024-10-17

**Authors:** Katarzyna Smółka, Lidia Perenc, Joanna Pelc, Leon Smółka, Konrad Szajnik

**Affiliations:** 1 Department of Child Neurology and Pediatrics, Clinical Regional Hospital No2, 35-301 Rzeszów, Poland; cm.katarzynasmolka@gmail.com (K.S.); joanna-pelc@o2.pl (J.P.); konrad.szajnik@gmail.com (K.S.); 2 Institute of Health Sciences, University of Rzeszów, 35-959 Rzeszów, Poland; 3 Department of Anatomy, Medical University of Silesia, 40-055 Katowice, Poland; s81350@365.sum.edu.pl

**Keywords:** thyrotoxic myopathy, ophthalmopathy, Graves’ disease, thyrotoxicosis, ptosis, differential diagnosis

## Abstract

**Background:** Myopathies encompass a wide range of diseases with diverse etiologies, courses, and prognoses, and can be either genetic or acquired in nature. One of the rare causes of acquired myopathies in children is hyperthyroidism. Ocular manifestations of hyperthyroidism include proptosis (exophthalmos) and widening of the palpebral fissure. Conversely, ptosis may indicate co-existing myasthenia or primary or secondary myopathy. **Methods**: This study presents a case of a 2-year-old child exhibiting both ocular disorders—each in one eye—along with features of proximal myopathy associated with undiagnosed thyrotoxicosis. **Results:** To our knowledge, this unique presentation of thyrotoxicosis in a young child has not been previously reported. After appropriate treatment for thyrotoxicosis, the child’s ocular and muscular symptoms showed improvement. **Conclusions**: Given that thyroid disorders can be a rare cause of both myopathy and ocular disorders in children, it is recommended that any child presenting with such symptoms undergo thyroid function screening tests.

## 1. Introduction

Myopathy refers to a variety of symptoms resulting from muscle damage, seen across a broad spectrum of diseases with varying causes, progressions, and prognoses. Myopathies can be a manifestation of genetic disorders or may be acquired. Thyrotoxicosis refers to a clinical condition characterized by abnormally high levels of circulating thyroid hormones—T3 (free triiodothyronine) and/or T4 (free thyroxine)—in the body, regardless of the underlying cause. It is often incorrectly used interchangeably with hyperthyroidism, which is a specific form of thyrotoxicosis caused by the excessive production of thyroid hormones by the thyroid gland itself [1].

Hyperthyroidism may be a very rare cause of acquired myopathy in children [2]. Ocular manifestations of hyperthyroidism include proptosis [3], while ptosis may indicate primary or secondary myopathy or may indicate co-existing myasthenia [4,5,6]. In thyrotoxicosis, symptoms of muscle and peripheral nerve damage develop rapidly, already in the early stages of endocrine disruption [7].

This study presents a case of a child exhibiting both ocular disorders and myopathy associated with undiagnosed thyrotoxicosis. To our knowledge, such a unique presentation of thyrotoxicosis in a toddler has not been previously reported.

## 2. Case Presentation

A 2-year-and-9-month-old girl, with an unremarkable perinatal history and typical early psychomotor development, was referred to the Department of Child Neurology and Pediatrics after confirmation of left eyelid drooping during the ophthalmologic examination. One month before hospitalization, the girl received treatment for bilateral conjunctivitis, during which tobramycin was administered for 12 days. Toward the end of the treatment, the mother noticed an episodic drooping of the left eyelid that occurred at different times of the day but most prominently in the afternoon. These episodes lasted up to 30 s, while asymmetry of the palpebral fissures persisted consistently throughout the day. The ophthalmologist examined the child and observed physiological hyperopia in both eyes and eyelid drooping on the left. Subsequently, the child was referred to ENT (ears, nose, and throat) and child neurology specialists. The ENT specialist recommended a sinus CT examination, which was not pursued due to the child’s lack of cooperation. Instead, amoxicillin–clavulanate and mometasone furoate were prescribed for 5 days. There was an improvement in nasal discharge with no change in the observed ocular symptoms; however, the child began snoring at night. Simultaneously, the child had chickenpox, managed symptomatically, with the last scab falling off two weeks before admission to the ward. Additionally, the girl’s mother observed tachycardia. A few days before admission, she underwent evaluation by a cardiologist, who recorded a heart rate of 130 bpm but found the structure and function of the heart to be normal otherwise. During the medical interview, the mother also reported frequent passing of stool of varying consistency, up to five times a day for the past few months. Given the presence of maternal celiac disease, it was excluded during the diagnostic evaluation of increased defecation in our patient. Despite undergoing potty training, the girl never signaled physiological needs.

**Figure 1:**
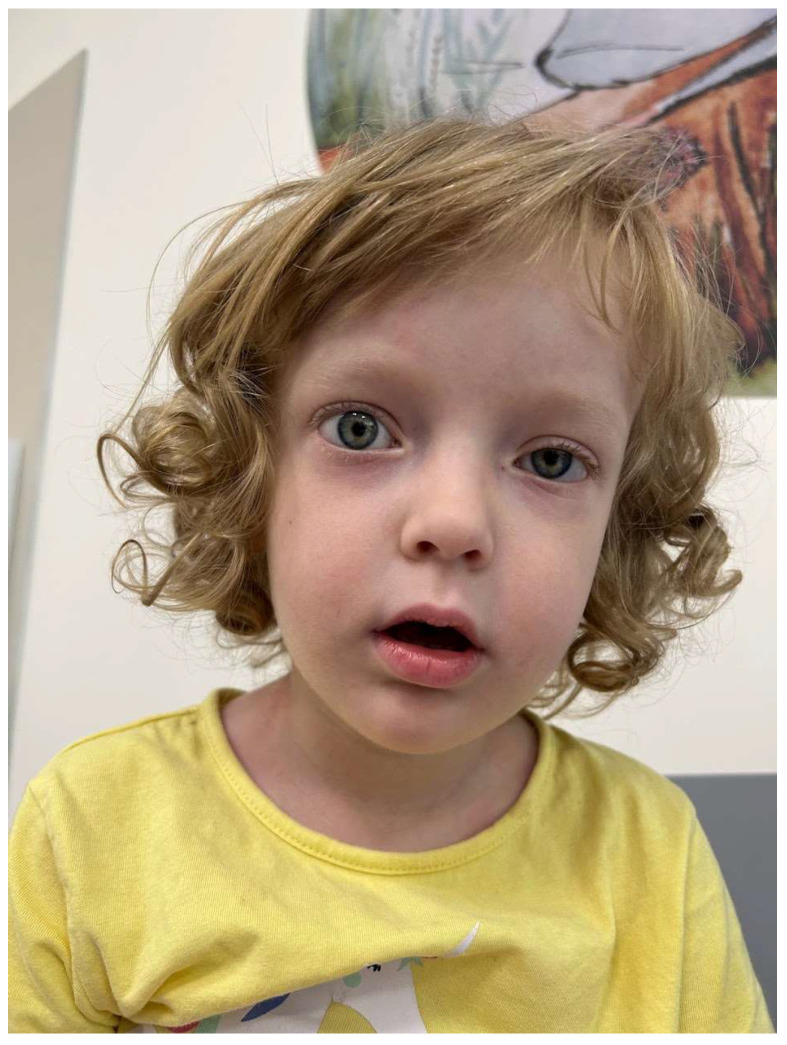
The myopathic face with unique ophthalmopathy—a slightly widened right palpebral fissure, a forward protrusion of the right eyeball, and evident ptosis on the left. Lip closure weakness is visible.

Upon admission, the girl exhibited hyperactivity, impatience, and a visible attention deficit. Physical examination revealed a notable height–weight discrepancy (97th percentile for height and 50–85th percentile for weight according to WHO growth charts) [8] and approximately 130 bpm tachycardia. Ocular abnormalities included a slightly widened right palpebral fissure, forward protrusion of the eyeball, and evident ptosis on the left. Lip closure weakness was also noted (Figure 1).

**Figure 2:**
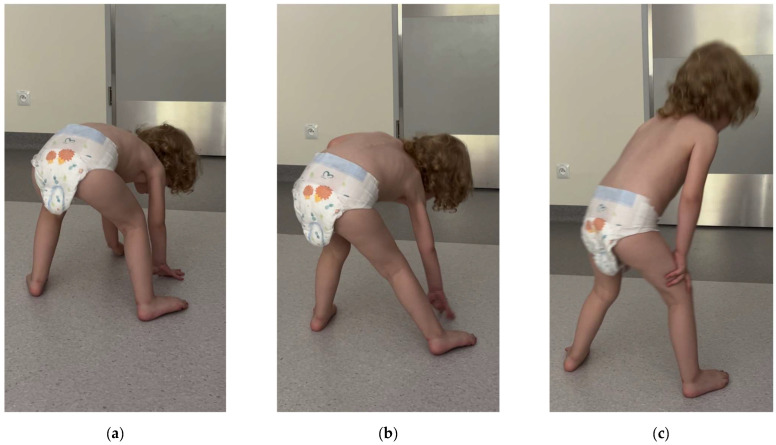
(**a**–**c**) Gower’s maneuver: using her hands and arms to “walk” up her body when standing up from the floor.

Neurological examination revealed visibly weakened muscle strength, particularly in the iliac girdle, along with decreased muscle tone. Deep reflexes in the upper extremities were vivid and symmetrical, while in the lower ones, they were difficult to induce. The girl’s gait was clumsy with a widened base, and she experienced difficulty standing up from a seated position on the floor, with a positive Gowers’ sign noted (Figure 2) (Video S1).

**Figure 3:**
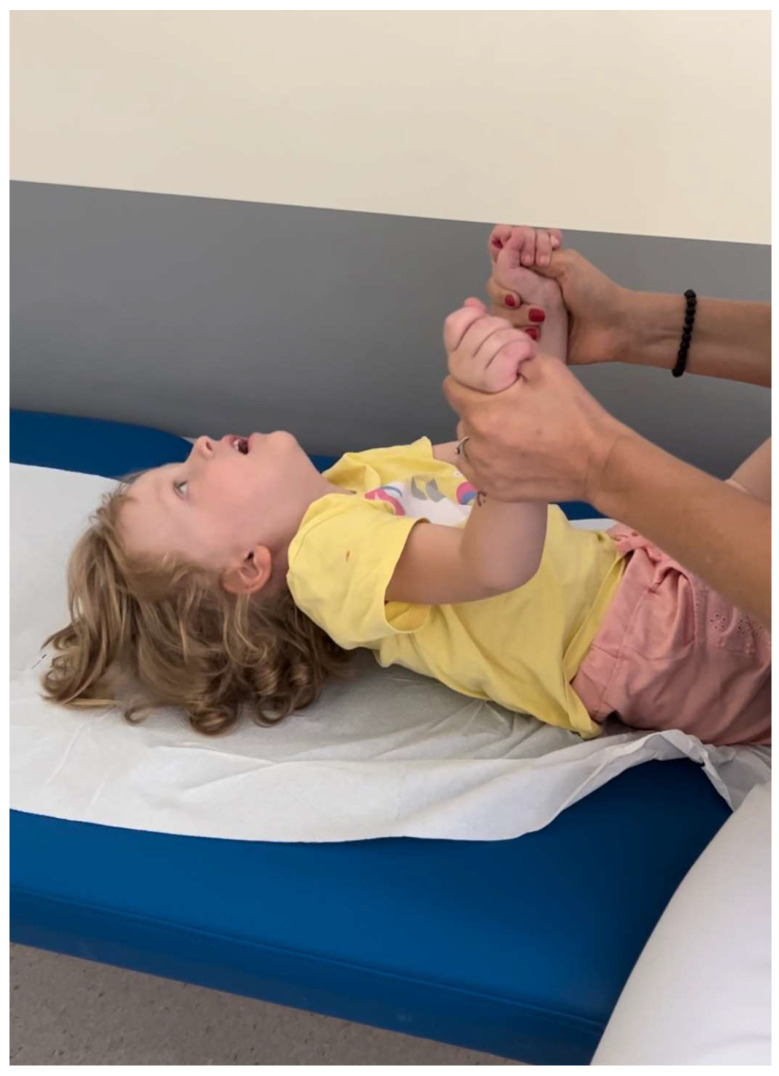
Head lag was observed during the examination.

In the supine position, she struggled to lift her head or raise her lower limbs above the base level (Figure 3).

Overall, the patient exhibited clinical features indicative of profound proximal myopathy, most prominently affecting the pelvic girdle and lower limbs.

Severe deviations from the norm were evident in the following laboratory tests: TSH (Thyroid-Stimulating Hormone) < 0.008 uIU/mL (reference range: 0.67–4.16 uIU/mL), fT3 > 20 pg/mL (reference range: 3.3–4.8 pg/mL), and fT4 > 6.0 mg/dL (reference range: 0.74–1.28 mg/dL). Other deviations included low levels of CK (creatine kinase)—48 U/L (reference range: 58–293 U/L) and indicators of microcytic anemia: HGB (hemoglobin) 10.7 g/dL (reference range: 11.5–14.5 g/dL), HCT (hematocrit) 32.1% (reference range: 35–45 dl), MCV (mean cell volume) 68.9 fl (reference range: 76–90.0 fl), and MICRO 27% (reference range: 0–5.0%).

Based on clinical and laboratory findings, the patient was diagnosed with hyperthyroidism, and further diagnostics were not performed, as fearing the onset of thyroid storm, the child was promptly referred to the Department of Pediatrics, Endocrinology, and Pediatric Diabetology for further assessment and treatment. Laboratory examinations confirmed elevated levels of free thyroid hormones, decreased levels of thyrotropic hormone, and significantly elevated titers of antibodies to thyroperoxidase and thyroid-stimulating hormone receptors. A thyroid ultrasound revealed features consistent with thyroid inflammation, numerous reactive cervical lymph nodes, and some enlargement. Additionally, a hypoechoic area measuring 2.5 × 1.6 mm^2^ was noted in the left lobe of the thyroid gland, without apparent vascularization. Based on these findings, a diagnosis of Graves’ disease was established.

**Figure 4:**
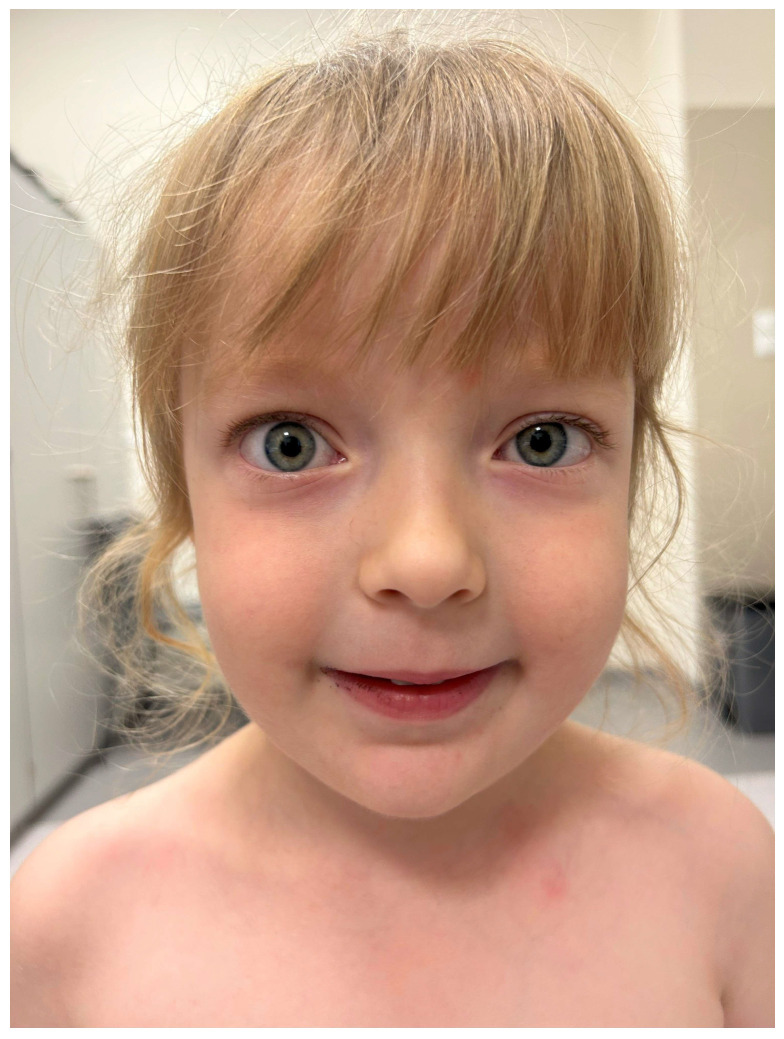
The patient’s face after 12 months of thiamazole treatment no longer exhibits a myopathic appearance. There is no weakness in lip closure or signs of ptosis. However, a slight protrusion of the right eyeball remains.

The patient was referred for a neurological follow-up, conducted one year after the initial diagnosis. Upon admission, the neurological examination revealed mild ocular abnormalities, including protrusion of the right eyeball, mild asymmetry in the eyelid creases, and no visible ptosis observed. The patient’s face no longer displayed a myopathic appearance (Figure 4).

Muscle strength was slightly reduced in the lower extremities, and muscle tone was generally low. Deep reflexes in both the upper and lower limbs were brisk and symmetrical. The gait was efficient, and the patient was able to stand up from the ground with only slight support on her right knee. Gowers’ sign was not observed. Head and trunk control were normal.

Laboratory tests (Table 1) showed low TSH at 0.013 uIU/mL (reference range: 0.67–4.16 uIU/mL), elevated fT4 at 1.830 mg/dL (reference range: 0.74–1.28 mg/dL), and increased TRAb (Thyroid-Stimulating Hormone Receptor Antibody) titers at 21.00 IU/l (reference range: <1.50 IU/mL) and TPO (Thyroid Peroxidase) antibodies at 209.90 IU/mL (reference range: <60 IU/mL), with negative TG (Thyroglobulin) antibodies. The patient was evaluated by an endocrinologist, who recommended increasing the dose of thiamazole. Circulating anti-AChR (Acetylcholine receptor) antibodies were borderline at 0.45 nmol/L (reference range: <0.40 nmol/L—negative, ≥0.40 to <0.50 nmol/L—borderline, ≥0.50 nmol/L—positive), and anti-MuSK (Muscle-Specific Kinase) antibodies were not detected, which did not confirm clinical suspicion of coexisting of myasthenia gravis. The creatine kinase level was within the normal range at 90 U/L (reference range: 68–293 U/L). An ENG neurographic examination showed normal conduction parameters in the examined nerves. Electromyography was not conducted due to a lack of cooperation. A follow-up thyroid gland ultrasound revealed enlargement with rounded edges, heterogeneous reduced echogenicity, and increased vascularization, consistent with autoimmune disease. A hypoechoic area/focal lesion measuring 3 × 3 mm^2^, with no apparent vascularization, was observed in the left lobe and required further monitoring. An MRI of the brain was normal but did show significant circular inflammatory thickening of the mucous membranes in the sphenoid sinuses and right maxillary sinus, with moderate thickening in the left maxillary sinus and ethmoid sinuses. The MRI of the eye orbits did not show pathology. The child was assessed by an ENT specialist, who determined that the child needed to undergo an adenoidectomy and myringotomy procedure.

## 3. Discussion

Thyroid hormones play a vital role in the functioning of the body, contributing significantly to growth and development throughout childhood and adolescence [9]. Hyperthyroidism, which comprises approximately 15% of pediatric thyroid disorders, is predominantly attributed to autoimmune hyperthyroidism, known as Graves’ disease, which accounts for 96% of cases [10,11]. The incidence of Graves’ disease among pediatric patients ranges from 0.1 to 3 cases per 100,000 children [11,12], often occurring in conjunction with other autoimmune conditions within the family or in the same patient [13]. Its prevalence increases with age, reaching a peak between 10 and 15 years, while cases of hyperthyroidism under the age of four are exceptionally rare, constituting only about 2% of all pediatric cases [14].

A study by Williamson et al. on 100,000 pediatric patients in the UK and Ireland identified weight loss (64%), fatigue (54%), behavioral changes (50%), and heat intolerance (47%) as the most common symptoms of acquired thyrotoxicosis, with 4.5% of cases presenting asymptomatically [10]. Physical examination typically reveals tachycardia, frequent bowel movements, warm moist skin, proximal muscle weakness, fine hand tremors, and goiter. Growth acceleration and advanced bone age may also be observed in children and adolescents [13]. Furthermore, up to one-third of pediatric patients may exhibit mild Graves’ ophthalmopathy [14]. Delayed diagnosis is common, as symptoms can be misinterpreted as behavioral disorders, respiratory problems, or cardiac arrhythmias [15].

Thyroid hormones play a crucial role in neurological and neuromuscular function, affecting skeletal muscle activity. Most individuals with thyrotoxicosis experience some degree of muscle weakness [2]. Duyff et al. reported that approximately 67% of individuals with newly diagnosed hyperthyroidism and 75% with hypothyroidism display muscle weakness [6]. The first case report of chronic thyrotoxic myopathy in children was published in 1974 [16], though the prevalence of thyrotoxic myopathy in the pediatric population remains unclear, with limited published reports [17,18,19,20,21,22,23]. The reported cases involved children aged between 2.5 and 11 years. The symptoms primarily included proximal muscle weakness, as well as additional manifestations such as proptosis, joint pain, weight loss, tachycardia, height–weight discrepancy, and, in one case, hypokalemic paralysis causing death. The most comparable clinical presentation of thyrotoxicosis in a 2.5-year-old boy, as reported by [20], included symptoms such as hypotonia, severe proximal muscle weakness primarily affecting the lower limbs, gait disturbances, and growth abnormalities. Additionally, tachycardia (120 bpm) and mild psychomotor delay were noted. Thyroid function tests revealed elevated levels of both bound and free thyroid hormone fractions, along with significantly suppressed TSH levels. A notable distinction, however, was in the ocular manifestations: while the Italian case involved bilateral exophthalmos, our patient exhibited unilateral ptosis and unilateral exophthalmos, affecting different eyes. We included an interesting case of Graves’ disease presenting with intermittent bilateral ptosis [24], predominantly affecting one eye, without associated myopathy, and with no evidence of myasthenia gravis. The biochemical findings varied, with abnormal levels of thyroid hormones. The treatment mainly involved the administration of antithyroid medications, such as carbimazole or methimazole, and, in one case, propranolol was also used. Muscle strength recovery varied depending on the case, ranging from 3 months to 6 years, depending on the severity of the disease and the treatment regimen. However, direct comparisons between the case studies are challenging due to variations in the laboratory tests used and the incomplete information on treatment regimens in some instances. The available data for these cases have been compiled and organized in Table 2.

Skeletal muscle expresses type 2 deiodinase, and through this enzyme, the muscle receives intracellular T3 from the precursor T4, with an adequate intracellular concentration of T3 enabling the physiological process of contraction and relaxation of muscle fibers [25].

The myopathy’s pathomechanisms vary depending on whether they are due to hyperthyroidism or hypothyroidism. In hypothyroidism, there is a global inhibition of major oxidative pathways and mitochondrial respiratory chain dysfunction. T3 deficiency leads to decreased glycogenolytic activity, resulting in accumulation of glycogen in muscles [26]. Moreover, T3 influences the transcription of numerous genes involved in muscle function, such as MyoD (Myoblast Determination protein 1), which regulates muscle cell regeneration and proliferation. Hypothyroidism causes atrophy and loss of type 2 (fast-twitch) muscle fibers, as well as hypertrophy of type 1 (slow-twitch) fibers [27]. In contrast, in hyperthyroidism, thyroid hormones increase the basal metabolic rate and promote mitochondrial oxidative uncoupling, leading to reduced muscle energy efficiency. This results in muscle weakness and fatigue [28]. Excess thyroid hormones can directly damage muscle fibers, leading to rhabdomyolysis [7]. Histological studies in patients with thyrotoxic myopathy have shown structural changes in muscles, such as muscle fiber atrophy, inflammatory infiltrates, and an increase in the number of nuclei in muscle cells [21].

Clinical symptoms of thyrotoxic myopathy include weakness in the proximal muscles, atrophy, and wasting, typically manifesting with an insidious onset and a slowly progressive course [21]. Muscle weakness primarily affects the pelvic and shoulder girdles, though all muscles may be involved to some extent [29,30]. Patients lacking typical hyperthyroidism symptoms can be challenging to diagnose early or may be mistaken for having primary myopathy, especially limb-girdle myopathy.

In our patient, pronounced proximal myopathy was observed, along with indications of weakening in distal, core, and face muscles. Due to the child’s hyperactivity, her mother did not recognize this weakness; instead, she perceived her as physically agile. Measurement of creatine kinase (CK) levels can aid in diagnosis, as CK levels are typically normal or decreased in chronic thyroid myopathy, contrasting with the elevated levels seen in primary myopathy [31].

Prakash et al. found a significant increase in CK levels in hypothyroid patients with low serum T3, with the opposite pattern observed in hyperthyroid patients. In hyperthyroidism, there was a negative correlation between CK activity and disease duration [32]. Excessive thyroid hormone levels seem to reduce CK activity and the contents of creatine and phosphate in skeletal muscle [21]. The serum creatine kinase (CK) level in our patient was found to be low.

The most common causes of hyperthyroidism in youth, Graves’ and Hashimoto’s diseases, are autoimmune [7,10,11]. Subsequent laboratory tests and thyroid sonography performed at the Department of Pediatrics, Endocrinology, and Pediatric Diabetology confirmed that the thyrotoxicosis in our patient was autoimmune. Our patient demonstrated nonspecific ocular symptoms. The right eye exhibited signs of Graves’ ophthalmopathy, such as exophthalmos, while the left showed intermittent ptosis. This symptom may indicate the onset of facial myopathy or a manifestation of co-existing myasthenia gravis. Due to the young age of our patient, assessing the presence of diplopia was challenging.

Mild forms of myasthenia gravis, particularly the ocular form, can also be seen in patients with autoimmune hyperthyroidism [7]. It is important to note that myasthenia gravis can be associated with hyperthyroidism in the course of differential diagnosis and subsequent treatment [33]. The prevalence of hyperthyroidism in children and adolescents with myasthenia gravis may be as high as 8% [34]. Oculomotor abnormalities are evident in both conditions: intermittent ptosis and associated diplopia are characteristic of myasthenia gravis, while exophthalmos and proptosis are hallmarks of Graves’ disease. Ophthalmopathy linked with Graves’ disease can be distinguished from myasthenia gravis by the presence of static symptoms rather than fluctuating ones [34]. However, the coexistence of ocular myasthenia in a patient with Graves’ disease is uncommon, with only a few cases reported to date [35], and sporadic reports have been documented in pediatric patients [6]. Perlman et al. reported a child with intermittent ophthalmoplegia and fluctuating ptosis of both eyes and facial weakness whose evaluation did not reveal evidence of myasthenia gravis but did reveal hyperthyroidism secondary to Graves’ disease. The authors suggest that symptoms commonly associated with myasthenia gravis can instead be caused by Graves’ disease and resolve with treatment of the endocrinopathy alone [24]. The diagnosis of coexisting ocular myasthenia gravis and thyroid eye disease may be difficult [36]. However, we did not find confirmation of coexisting myasthenia gravis in our patient. To our knowledge, there are currently no reports in the literature of exophthalmos in one eye combined with concurrent ptosis in the other eye due to thyroid myopathy. This clinical presentation warrants further investigation to identify the underlying pathomechanisms.

Our patient exhibited a variety of symptoms characteristic of hyperthyroidism, including anxiety, attention-deficit disorder, tachycardia, frequent bowel movements, proximal muscle weakness, excessive growth, and ophthalmopathy. Throughout her diagnostic journey, which lasted several months, she was evaluated by pediatric specialists in psychology, cardiology, gastroenterology, ophthalmology, and ENT. Each specialist focused on symptoms related to their field, leading to a fragmented understanding of her overall clinical picture and the delay in diagnosis. At follow-up, although TSH levels remained outside the normal range, general symptoms of hyperthyroidism, such as tachycardia and frequent bowel movements, were not observed. The child’s behavior and attention span had improved. While ocular symptoms persisted, they were relatively unremarkable, consisting primarily of slight bulging of the right eye and minimal ptosis on the left. Significantly, there was marked improvement in muscle tone and strength, aligning with literature reports on the remission of signs during treatment [21].

The implications of this case for clinical practice are profound, particularly in the way it highlights the need for expanded diagnostic protocols and improved treatment approaches for pediatric patients with similar complex symptoms. Firstly, this case emphasizes the necessity of considering thyroid dysfunction, such as thyrotoxicosis, as a differential diagnosis when children present with unexplained myopathy and/or ocular abnormalities. Thyroid function screening should become a standard component of the diagnostic workup for children presenting with muscle weakness, especially when symptoms involve proximal myopathy or ophthalmopathy, as early recognition can prevent misdiagnosis or delayed treatment. Furthermore, this case illustrates the importance of interdisciplinary collaboration in managing complex cases. Involvement from pediatric neurology, endocrinology, ophthalmology, and other specialties can help integrate disparate symptoms into a cohesive diagnosis, ensuring that no aspect of the patient’s condition is overlooked.

At the conclusion of this discussion, we would like to present our proposed approach for differentiating disease entities, including those with etiological heterogeneity, that cause ptosis (Table 3). This symptom may present unilaterally or bilaterally and can initially appear on one side before progressing to both. The onset of ptosis-related diseases or syndromes can vary significantly, with the condition manifesting either congenitally or being acquired later in life. Notably, congenital ptosis is not always genetically determined, and acquired ptosis can have a genetic basis. The disease progression may be acute, chronic, progressively worsening, or recurrent. In cases of hereditary congenital ptosis, ptosis may present as an isolated symptom. However, in other instances, it coexists with additional symptoms, the analysis of which is crucial for accurate diagnosis. Recognizing these co-occurring symptoms and understanding the factors underlying specific diseases can facilitate the selection of appropriate diagnostic methods and aid in the differentiation process. Thus, the choice of treatment is closely aligned with the identified etiological factor, or, in the absence of such a determination, the idiopathic nature of the disease [4,18,23,37,38,39,40,41,42,43,44,45,46,47,48,49,50,51,52,53,54,55,56,57,58,59,60,61,62,63,64,65,66,67,68,69,70,71,72,73,74,75,76,77,78,79,80,81,82,83,84,85,86,87,88,89,90].

In our patient, ptosis was unilateral, acquired, and chronically progressive, co-occurring with Gowers’ sign, which suggested the presence of myopathy. The concurrent systemic symptoms and elevated TSH levels indicated a potential autoimmune etiology. While the literature offers a high-quality example of differentiating ptosis [4], it does not include thyrotoxic myopathy. Our clinical case suggests considering Graves’ disease in the differential diagnosis of ptosis.

## 4. Conclusions

Interdisciplinary collaboration is fundamental in diagnosing complex cases, as it ensures a comprehensive evaluation of the patient’s condition. Each specialist, whether in neurology, endocrinology, pediatrics, or other relevant fields, must not only concentrate on symptoms within their domain, but also remain attentive to signs outside their area of expertise. Detailed history-taking and considering the broader clinical presentation can help in identifying the underlying cause of the patient’s symptoms. A narrow focus on specialty-specific symptoms risks overlooking key aspects of the patient’s condition, which can delay diagnosis and treatment. A more integrative and holistic approach is essential for reducing diagnostic delays and providing optimal patient care.

Thyroid disorders are common, and every pediatric specialist’s attention to physical examination findings aids in early diagnosis and treatment. Thyrotoxicosis may be a rare cause of myopathy, so a child with muscle weakness undergoing assessment for neuromuscular disease should always receive thyroid function screening tests as part of their evaluation.

## Figures and Tables

**Table 1:** The test results and pharmacotherapy documented at various stages: at the moment of diagnosis, throughout the treatment in the Department and Outpatient Pediatric Endocrinology Clinic, and during neurological follow-up in the Department of Child Neurology and Pediatrics.

		Department of Child Neurology and Pediatrics	Pediatric Endocrinology Department			Pediatric Endocrinology Outpatient Clinic										Department of Child Neurology and Pediatrics
	Units & Reference Range/Date	13.07.2023	18.07.2023	21.07.2023	25.07.2023	16.08.2023	30.08.2023	27.09.2023	25.10.2023	22.11.2023	13.12.2023	21.02.2024	27.03.2024	24.04.2024	22.05.2024	14.06.2024
Laboratory Tests Results	TSH µIU/mL <0.670–4.16>	<0.008 ↓	<0.008 ↓	<0.008 ↓	<0.008 ↓	<0.008 ↓	<0.008 ↓	<0.008 ↓	<0.008 ↓	<0.008 ↓	0.079 ↓	<0.008 ↓	0.956 N	2.064 N	0.499 ↓	0.013 ↓
	FT3 pg/mL <3.3–4.8>	>20.0 ↑	11.34 ↑	8.94 ↑	6.66 ↑	4.54 N	5.52 ↑	9.57 ↑	8.12 ↑	4.08 N	3.91 N	6.71 ↑	3.44 N	3.87 N	6.15 ↑	–
	FT4 ng/dL <0.74–1.28>	>6.0 ↑	2.74 ↑	2.03 ↑	1.65 ↑	0.97 N	1.07 N	1.83 ↑	1.51 ↑	0.77 ↓	0.62 ↓	1.47 ↑	0.51 ↓	0.65 ↓	1.0 N	1.83 ↑
	Hb gr/dL <11.5–14.5>	10.7 ↓	11.3 ↓	11.1 ↓	11.1 ↓	12.0 N	11.6 N	12.1 N	11.4 ↓	12.6 N	13.0 N	11.7 N	13.0 N	12.1 N	12.5 N	12.8 N
	MCV fl <76–90>	68.9 ↓	69.2 ↓	69.6 ↓	70.3 ↓	70.5 ↓	69.9 ↓	71.4 ↓	70.6 ↓	72.1 ↓	74.0 ↓	75.4 ↓	75.9 ↓	76.0 N	75.2↓	74.4 ↓
	p/c anti TPO IU/mL <60>	177.9 ↑	–	–	–	–	–	–		–	–	–	–	–	–	209.9 ↑
	TRAb IU/L <1.5>	>30.0 ↑	–	>30.0 ↑	–	>30.0 ↑	–	>30.0 ↑	–	–	–	–	27.8 ↑	–	–	21.0 ↑
	CK U/L <68–293>	40.8 ↓	–	–	–	–	–	–	–	–	–	–	–	–	–	90.0 N
	IgA g/L <0.6–2.1>	0.48 ↓	–	–	–	–	–	–	–	–	–	–	–	–	–	0.83 N
Pharmacotherapy	Thyrozol (tiamazol) <mg>	–	–	–	–	1.25–0–1.25	1.25–0–1.25	2.5–0–2.5	2.5–0–2.5	2.5–0–2.5	1.25–0–1.25	2.5–0–2.5	1.25–0–1.25	1.25–0–0	1.25–0–0	2.5–0–2.5
	Propranolol <mg>	–	–	–	5–2.5–2.5	2.5–0–2.5	2.5–0–0	2.5–0–0	2.5 mg as needed in case of tachycardia							

↑—above reference range, ↓—below reference range, N—in the reference range.

**Table 2:** Cases of thyrotoxic myopathy in children.

Research	Age	Sex	Symptoms	Laboratory Test Results	Treatment	Muscle Strength Recovery
Johnston, D.M. (1) [16]	6	M	Weakened muscle strength, double proptosis, family history of thyrotoxicosis	T3 uptake 76%, T4: 17.7 ug/100 mL (reference range 4.5–13.6), TSH: no data, PBI: 13.6 ug/100 mL (reference range 3.8–8.0), CK: 16 mIU/mL (reference range > 140)	Carbimazole 5 mg 3×/day	Full recovery after 3 months
Johnston, D.M. (2) [16]	10	M	Weakened muscle strength, weight loss, slight proptosis	T3 uptake 77%, T4: no data, TSH: no data, PBI: 12.8 g/100 mL, CK: 43 mIU/mL	Carbimazole 10 mg 2×/day	Full recovery after 4 months
Radetti, G. [18]	8	M	Seizure with coma, weakened muscle strength	T3/T4: no data, TSH: no data, CK: 69 IU/L (reference range < 170)	Methimazole 7.5 mg/day	Full recovery after 6 years
Greco, F. [20]	2.5	F	Weakeness of proximal muscle, gait abnormality, weight loss, anxiety, exophthalmos, confirmed Graves’ disease	T3—30 pg/ml (reference range: 3.05–5.35 pg/mL), T4—10.85 ng/dL (reference range: 0.71–1.85 pg/mL), TSH—0.01 mcU/mL (v.n. 0.5–3), CK—normal (no more data)	Methimazole 7 mg/day for 15 days, then reduced to 1.5 mg/day	Improvement of gait and muscules strenght after 4 weeks therapy (no further follow up)
Drash, P. [22]	2.5	M	Weakened muscle strength, proptosis, joint pain	T3/T4: no data, TSH: no data, PBI: 16.7 mg/100 mL, CK: no data	Propylthiouracil 100 mg 3×/day	Full recovery after approximately 5 years
Satam, N. [23]	10	F	Respiratory failure, weakeness of proximal muscle, fever, exopthalmos, lid lag	T3—6.39 pg/mL (reference range: 2.3–4.2 pg/mL), T4—3.00 ng/mL (reference range: 0.98–1.76 ng/dL), TSH < 0.002 μIU/mL (reference range 0.35–5.50 μIU/mL), CK: no data	Propranolol (no more data).	Fatal case. A post-mortem biopsy striated muscle showed findings suggestive of acute thyrotoxic myopathy.
Perlman, S.J. [24]	5	M	bilateral ptosis, more pronounced in one eye, dysconjugate eye movements, intermittent diplopia, fatigue, no muscle weakness	T4—2.64 ng/mL (reference range: 0.8–1.8 ng/dL), TSH 0.01 μIU/mL (reference range 0.35–5.50 μIU/mL), thyroid peroxidase antibodies 1281 IU/mL (reference range 0–9) thyroid-stimulating immu- noglobulin 217% (reference range 0–129), CK: no data	Methimazole (no more data).	Normalization of extraocular movements, resolved ptosis, and normal acial strength after 3 months and at 11 months follow up.

T3—Triiodothyronine, T4—Thyroxin, TSH—Thyroid Stymulating Hormone, PBI—Protein-bound iodine, CK—Creatine Kinase.

**Table 3:** Differentiation of disease entities causing ptosis.

Name		Unilateral/Bilateral	Other Symptoms	Etiology
Congenital ptosis				
Hereditary congenital ptosis [37]		Unilateral/Bilateral	lack	mutation in PTOS1, PTOS2 gene
Congenital ptosis and other congenital defects [38]		Bilateral	facial dysmorphism, strabismus, camptodactyly, syndactyly, broadening of the first rays, intellectual disability, autism spectrum disorder	loss of ZFHX4 gene in submicroscopic deletion of 8q21.11
Muenke syndrome [39]		Unilateral/Bilateral	syndromic craniosynostosis, significant phenotypic variability, midfacial retrusion, strabismus, hearing loss, developmental delay	mutation in FGFR3 gene
Saethre-Chotzen syndrome [40]		Unilateral/Bilateral	syndromic craniosynostosis, low frontal hairline, strabismus, tear duct stenosis, brachydactyly, partial cutaneous syndactyly of digits 2 and 3 of the hand, duplicated distal phalanx of the hallux, intelligence is normal, developmental delay	mutation in TWIST1 gene
Duane retraction syndrome [41,42]		Unilateral	primary gaze position esotropia with limitation of abduction (abducens nucleus and nerve are absent or hypoplastic, and a branch of the oculomotor nerve innervates the lateral rectus muscle, Type 1), adduction paresis (Type 2), abduction and adduction paresis (Type 3)	mutation in CHN1, MAFB, SALL4 genes
Blepharophimosis ptosis epicanthus inversus syndrome [43,44]		Bilateral	telecanthus (Types 1–2), and premature ovarian failure (Type I), others: blepharophimosis, epicanthus inversus, congenital cataract	mutation in FOXL2 gene
Congenital fibrosis of the extraocular muscles [45]		Bilateral	Marcus Gunn jaw winking, bilateral exotropic ophthalmoplegia with pupillary abnormalities, mental abnormalities hand oligodactyly, globe retraction with synergistic divergence (Type 1–5)	mutation in KIF21A, PHOX2A, TUBB3, TUBB2B, TUBA1A, ECEL1, COL25A1 genes
Marcus Gunn phenomenon [46,47]		Unilateral/Bilateral	congenital facial synkinesis known as jaw-winking or pterygoid-levator synkinesis, raising of the affected eyelid is synchronous and proportionate to the opening of the mouth., rare: cleft lip and cleft palate	not identified. A branch of the trigeminal nerve congenitally misdirected
Congenital facial palsy [48]		Unilateral Charles Bell’s sign, rarely bilateral	unilateral facial weakness/paralysis of the facial muscles, ipsilateral abducens nerve palsy (Moebius syndrome), rarely bilateral	brain stem ischemia, infectious agent, perinatal injury teratogenic factors (hyperthermia, thalidomide, ergotamine, chorionic villus sampling), hereditary
Congenital myasthenic syndromes [49]		Bilateral	abnormal muscle fatigue may be weakly expressed, in newborn respiratory failure with episodes of apnea, cyanosis, generalized weakness or early onset in childhood, delayed motor milestones, weakness of facial, ocular bulbar, and axial muscle, scoliosis in older patients	defects in molecules expressed at the neuromuscular junction, mutations in 35 genes have been reported., most common in CHAT, CHRNE, COLQ, DOK7, GFPT1, and RAPSN genes
Turner syndrome [50]		Bilateral	webbed neck, shield chest, epicanthal folds, short stature, gonadal dysgenesis, infertility, congenital and acquired cardiovascular anomalies, specific cognitive, psychosocial phenotype	karyotype 45, X
Noonan syndrome [51,52]		Bilateral	facial dysmorphism, webbed neck, chest deformity, congenital heart defects, cardiomyopathy, multiple lentigines, increased risk of developing tumors in childhood, intellectual disability, behavioral problems	mutations in PTPN11, SOS1, RAF1, RIT1, LZTR1 genes
Smith-Lemli-Opitz syndrome [53,54]		Bilateral	microcephaly, cleft palate, cardiac defects, underdeveloped external genitalia, post-axial polydactyly, 2 to 3 toe syndactyly intellectual deficit, behavioral problems, elevated 7-dehydrocholesterol	mutation in DHCR7 gene
Rubinstein-Taybi syndrome [55,56,57]		Bilateral	microcephaly, down slanting palpebral fissures, strabismus, beaked nose, broad and short thumbs and first toes, short stature, seizures, intellectual disability and behavioral characteristics	microdeletion of 16p13.3 or microdeletion of 22q13.2
Congenital & acquired ptosis				
Horner syndrome [58,59,60,61]		Unilateral	ipsilateral miosis, enophthalmos and anhidrosis, may present in association with a lighter color of the iris of the affected eye or contralateral hemifacial flushing and ipsilateral hypohidrosis (Harlequin syndrome)	results from an interruption of the oculosympathetic pathway, perinatal brachial plexus injury, internal carotid artery agenesis, dissection, neuromyelitis optica, postviral damage, cervical disc herniation, osteochondroma of the first rib, neuroblastomas, other tumors
Acquired ptosis				
Chronically progressive with possible exacerbations	Progressive external ophthalmoplegia (PEO) [62]	bilateral (initially may be unilateral)	isolated PEO or PEO plus syndromes: mitochondrial neuro gastrointestinal encephalomyopathy (MNGIE), sensory ataxic neuropathy dysarthria ophthalmoplegia (SANDO)	deletions of mitochondrial DNA
	Kearns-Sayre syndrome [63]	bilateral (initially may be unilateral)	pigmentary retinopathy, cardiac conduction abnormalities, cerebellar ataxia, deafness, endocrinopathies	deletions of mitochondrial DNA
	Oculopharyngeal muscular dystrophy [64]	Bilateral	late-onset intractable myopathy, ophthalmoplegia without diplopia, dysphagia, dysarthria and proximal limb weakness	dynamic mutation in PABPN1 gene
	Myotonic dystrophy [65,66,67,68]	Bilateral	in newborn: hypotonic and difficulties with breathing and feeding, clubfeet, later onset: motor symptoms, myotonia, muscle weakness, social and learning difficulties, hypersomnia, cardiac symptoms or cataracts (Type 1), muscle weakness, myalgia, myotonia, tremor, early and severe cardiac involvement, diabetes, cataracts (Type II)	dynamic mutation in DMPK gene (Type I), dynamic mutation in CNBP gene (Type II)
	Myasthenia gravis [69,70]	bilateral (initially may be unilateral)	abnormal muscle fatigue, diplopia, dysphagia, dysphonia or dysarthria, mastigatory impairment, dyspnea, asthenia, weakness of the cervical muscles and of the extremities	autoimmune
	Graves-Basedow’s disease [13,34,71]	Unilateral/Bilateral	exophthalmos, myopathy, tachycardia, difficulty in breathing, diarrhea, anemia, convulsions, coma, thyrotoxic-hypokalemic periodic paralysis	autoimmune
	Lambert-Eaton myasthenic syndrome [72]	Bilateral (initially may be unilateral)	classic clinical triad of proximal weakness, autonomic dysfunction, and areflexia	autoimmune
Acute / Chronic	Bell’s palsy (Idiopathic peripferal facial nerve palsy) [73]	Unilateral Charles Bell’s sign, rarely bilateral	unilateral facial weakness/paralysis of the facial muscles	idiopathic
	Oculomotor nerve palsy [74,75,76,77,78,79]	Unilateral	diplopia, strabismus divergens, dilated pupil with abolished reaction to light, impaired upward, adducted and partially downward eye movement, retrobulbar and frontal pain may occur	infection, demyelination, vasculopathy, compressive processes (posterior communicating artery aneurysms, proliferative diseases, hydrocephalus, cavernous sinus thrombosis), sphenoiditis
Acute	Peripheral facial nerve palsy [80,81,82,83]	Unilateral Charles Bell’s sign, rarely bilateral	unilateral facial weakness/paralysis of the facial muscles, rarely bilateral	infection (Lyme disease), demyelination, vasculitis (Melkersson-Rosenthal syndrome), compressive processes (cerebellopontine angle tumor, neurofibromatosis)
	Acute botulism [84,85]	Bilateral	diplopia, blurred vision, dry mouth, dysphagia, dysphonia, dysarthria	Clostridium botulinum
	Miller-Fisher syndrome [86,87]	Bilateral	external ophthalmoplegia, ataxia, and areflexia	autoimmune
	Ophthalmoplegic migraine [88]	Unilateral	external ophthalmoplegia, elimination of pupil reaction to light	inflammatory vasculopathy
	Nonspecific orbital inflammation [89]	Unilateral	periorbital edema, palpable mass, dacryoadenitis, oculomotor myositis, systemic signs: headache, emesis, anorexia, lethargy, fever, additionally, there are likely associations with iritis, uveitis, optic disc edema, peripheral eosinophilia	complex interaction of immunological dysregulation, viral triggers, and genetic predisposition
	Infiltration of blasts and formation of disease foci in the body (palpebre) outside the bone marrow in the course of leukemia (myeloid sarcoma) [90]	Unilateral	blood smear typical of leukemia	specific to a specific type of leukemia

CHAT—choline O-acetyltransferase, CHN1—chimerin 1, CHRNE—cholinergic receptor nicotinic epsilon subunit, CNBP—cellular nucleic acid-binding protein, COL25A1—collagen type XXV alpha-1 chain, COLQ—collagen like tail subunit of asymmetric acetylcholinesterase, DHCR7—7-dehydrocholesterol reductase, DMPK—dystrophia myotonica protein kinase, DNA—deoxyribonucleic acid, DOK7—docking protein 7, ECEL1—endothelin converting enzyme like 1, FGFR3—fibroblast growth factor receptor 3, FOXL2—forkhead box protein l2, GFPT1—glutamine-fructose-6-phosphate transaminase 1, KIF21A—kinesin family member 21a, LZTR1—leucine zipper like post translational regulator 1, MAFB—V-maf musculoaponeurotic fibrosarcoma oncogene homolog B, PABPN1—polyadenylate-binding nuclear protein 1, PHOX2A—paired mesoderm homeobox protein 2A, PTOS1—ptosis hereditary congenital 1, PTOS2—ptosis hereditary congenital 2, PTPN11—protein tyrosine phosphatase non-receptor type 11, RAF1—proto-oncogene serine/threonine-protein kinase, RAPSN—receptor associated protein of the synapsę, RIT1—guanosine-5′-triphosphate-binding protein, SALL4—spalt like transcription factor 4, SOS1—‘son of sevenless’ guanine nucleotide exchange factor 1, TUBA1A—tubulin alpha 1a, TUBB2B—tubulin beta 2B class IIb, TUBB3—tubulin beta 3 class III, TWIST1—twist-related protein 1, ZFH4—zinc finger homeobox 4.

## Data Availability

The original contributions presented in the study are included in the article and Supplementary Materials, further inquiries can be directed to the corresponding author.

## Supplementary Materials

The following supporting information can be downloaded at: https://www.mdpi.com/article/10.3390/jcm13206180/s1, Video S1: The Gowers’ sign.
